# Sound-Based Tool Wear Classification in Turning of AISI 316L Using Multidomain Acoustic Features and SHAP-Enhanced Gradient Boosting Models

**DOI:** 10.3390/ma19050861

**Published:** 2026-02-25

**Authors:** Savaş Koç, Mehmet Şükrü Adin, Ramazan İlenç, Mateusz Bronis, Serdar Ekinci

**Affiliations:** 1 Engineering Faculty, Batman University, Batman 72100, Turkey; 2 Besiri OSB Vocational School, Batman University, Batman 72060, Turkey; 3 Department of Machine Design and Machining, Kielce University of Technology, 25314 Kielce, Poland; mateuszbronisck@gmail.com; 4 Faculty of Engineering and Architecture, Bitlis Eren University, Bitlis 13100, Turkey; sekinci@beu.edu.tr

**Keywords:** tool wear classification, acoustic signal analysis, multidomain feature extraction, gradient boosting models, LightGBM, XGBoost, CatBoost, SHAP-based feature selection

## Abstract

Reliable tool-wear monitoring is essential for maintaining machining quality and preventing unscheduled downtime in manufacturing. This investigation presents a sound-based classification framework for identifying wear states in the turning of AISI 316L stainless steel using advanced gradient-boosting models. Acoustic signals were recorded under constant cutting parameters to eliminate process-induced variability, and each recording was divided into standardized 2 s segments. A total of 540 multidomain features—including RMS, ZCR, spectral descriptors, Mel-spectrogram statistics, MFCCs and their derivatives, and discrete wavelet energies—were extracted to capture both stationary and transient characteristics of tool–workpiece interactions. Feature selection was performed using a three-stage pipeline comprising Boruta, LASSO, and SHAP analysis, resulting in a compact subset of highly informative descriptors. LightGBM, XGBoost, and CatBoost classifiers were trained using stratified 10-fold cross-validation across three wear states: Unworn, Slight wear, and Severe wear. LightGBM and XGBoost achieved the best performance, with mean accuracies above 0.96 and strong PRC–AUC and ROC–AUC values (0.98–1.00). Although Slight wear remained the most difficult class due to its transitional acoustic characteristics, all models showed clear separability for Unworn and Severe wear conditions. The results confirm that boosted decision-tree methods combined with SHAP-enhanced feature selection provide an effective, low-cost, and non-contact solution for tool-wear classification in 316L turning.

## 1. Introduction

In the present day, many products with different features and designs are manufactured in modern manufacturing industries. However, these manufactured products require many different processes, such as turning, milling, and drilling, for their end-use applications. One of the operations used to process manufactured products is turning. One of the most basic machining processes, turning, is essential for attaining accuracy and productivity in manufacturing industries [1,2,3]. When considering the usage intensity of steels in contemporary manufacturing industries, AISI 316L stainless steel stands out particularly among other steels. What distinguishes AISI 316L stainless steel from other steels is its particularly high strength and corrosion resistance, which creates challenging conditions during turning operations [3,4]. One of the most significant problems encountered is the excessive wear of cutting tools when machining these types of materials. High temperatures, high pressure, and high friction are the main causes of rapid cutting tool wear, according to previous investigations published in the literatüre [5,6]. Flank wear, microcrack, crater wear, adhesion, and oxidation formation are the most prevalent types of tool wear. Because of the constant contact between the tool’s lateral surface and the workpiece, flank wear is especially important. Therefore, keeping an eye on flank wear is crucial for maximizing machining productivity and prolonging tool life [1,7,8,9]. According to Del Risco-Alfonso et al. [10], AISI 316L stainless steel’s work-hardening propensity and adhesive qualities further hasten flank wear. In a similar way, Persson et al. [11] discovered that this type of wear is made worse by higher thermal loads. It has been demonstrated that tool wear lowers overall production efficiency, diminishes dimensional accuracy, and deteriorates surface finish [1,12]. Errors and production delays are also caused by ineffective tool wear monitoring techniques and operators’ propensity to wait to change tools until wear develops [13]. For many years, investigations have concentrated on real-time monitoring of tool wear in turning processes. On the other hand, high-quality processing of materials depends entirely on the operators’ expertise. Operators need to properly optimize the timing of tool replacements; replacing a worn tool can result in decreased dimensional accuracy, greater surface roughness, and higher energy consumption, while replacing a tool too quickly can create delays due to machine downtime. Therefore, limiting unplanned downtime, maintaining process continuity, and lowering maintenance costs all depend on early detection and precise classification of wear situations [1,14].

Although they offer great precision, traditional tool wear monitoring techniques like optical inspection, cutting force measurement, and vibration analysis frequently necessitate stopping the machining process, which makes them expensive and time-consuming [15,16,17,18]. The benefit of non-contact and real-time monitoring is provided by indirect systems, which use process data such as cutting force, vibration, temperature, and acoustic signals; nevertheless, the requirement for additional sensors might raise costs in industrial applications. Among these, vibration and acoustic sensors are susceptible to installation conditions despite their broad frequency range, while force sensors are very accurate but restrict the size of the workpiece. Also, the Hall effect-based current sensors need to be positioned outside the machine. Microphones, on the other hand, stand out as a useful substitute because they provide simple and adaptable installation without interfering with the equipment’s regular operation [19,20,21,22].

Due to its non-contact nature, low cost, and ease of integration into current machining environments, acoustic signal analysis has become a particularly attractive method for tool wear detection in recent years. Moreover, a number of investigations have revealed that machining sounds provide important information on the interactions between the tool and the workpiece, allowing for quick and accurate wear classification without interfering with production [23,24]. For instance, Zafar et al. [25] used sophisticated noise filtering to increase robustness in industrial settings, whereas Bhandari [26] claimed 90% classification accuracy utilizing Mel-frequency cepstral coefficients (MFCCs) and Mel-spectrogram features. When these investigations are carefully and comprehensively evaluated, they demonstrate that voice-based monitoring has high potential for non-invasive, affordable, and real-time wear detection. Furthermore, recently developed sound- and hybrid-sensing pipelines reported high accuracy in a variety of setups, including thermography-driven deep Convolutional Neural Networks (CNNs), force–vibration–acoustic emission (AE) with 1D CNN + TCN, ultrasonic microphone arrays and high-frequency microphones, spectrogram-based AE/microphone CNN, multi-sensor vibration–current–force with ResNet + Long Short-Term Memory (LSTM), explainability-aware SHapley Additive exPlanations (SHAP) + RF, parameter-driven Artificial Neural Networks (ANNs) on AISI steels, and sensor-fusion with Gradient Boosting (GBM) [12,20,23,27,28,29,30,31]. As is known, the rapid advancement of technology has significant and beneficial effects in many different fields. One area positively impacted by rapidly evolving technology is undoubtedly the significant advancements in the field of sensing [14]. Machine learning (ML) and Ensemble Learning (EL) have significantly enhanced TCM systems’ capacity to handle complicated, high-dimensional, and noisy input in parallel with advancements in sensing. Specifically, explainability strategies (like SHAP) improve operator trust and decision transparency, while ensembles like Extra Trees and Random Forest have demonstrated better generalizability to unforeseen machining situations than single models 3. Multimodal sensor fusion using GBM can surpass classical baselines, according to studies on metal and wood machining [30,31,32,33].

Previous investigations in the literature have revealed that machining machine parameters and material characteristics have an important effect on tool wear rates, which in turn influences overall productivity in the industry. Comparative studies of the acoustic signals produced by turning AISI 316L stainless steel are still scarce, nevertheless. The present investigation uses sophisticated gradient-boosting models to propose a sound-based classification framework for detecting wear situations in the turning of AISI 316L stainless steel.

## 2. Materials

### 2.1. Properties of AISI 316L Stainless Steel

**Figure 1:**
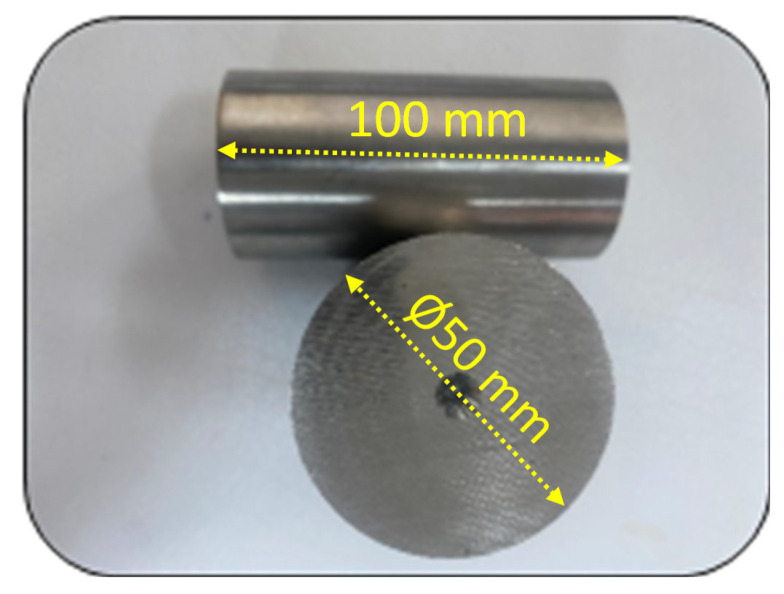
AISI 316L stainless steel used in turning.

In this research, AISI 316L stainless steels were used as workpiece materials, and their dimensions are shown in Figure 1. AISI 316L is an austenitic stainless steel with excellent corrosion resistance, suitable for industrial applications in chemical, marine, and medical sectors. Its tensile strength ranges from 485 to 620 MPa, with a minimum yield strength of 170 MPa, elongation between 40% and 60%, and a density of 7.99 g/cm^3^. The AISI 316L stainless steel specimens used in this study exhibit typical mechanical properties, including an ultimate tensile strength of 485–620 MPa, a yield strength of 170–290 MPa, a hardness of approximately 160 HV (150–200 HV range), and a fracture elongation of 35–60% in the annealed condition [34].

The chemical compositions of AISI 316L stainless steel are given in Table 1, consisting of key elements such as chromium (Cr), nickel (Ni), manganese (Mn), and carbon (C).

### 2.2. DNMG Tool Insert

**Figure 2:**
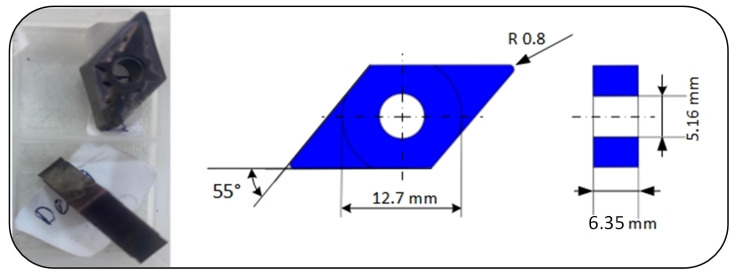
DNMG 150608-MA HS7225 carbide insert.

A CVD-coated carbide insert (DNMG 150608-MA, grade HS7225) was used for the turning experiments. As shown in Figure 2, the insert has a rhombic geometry with a 55° included angle and a negative rake angle, enabling double-sided use. Designed for high-speed machining of stainless and alloy steels, it offers high wear resistance and extended tool life [35].

The MA-type chipbreaker ensures effective chip control under medium to heavy cutting conditions. Geometrical and physical specifications are provided in Table 2. Since the study focuses exclusively on AISI 316L, the same cutting insert was used across all experiments to maintain consistency in wear patterns.

### 2.3. Captured Images and Sound Recorder

**Figure 3:**
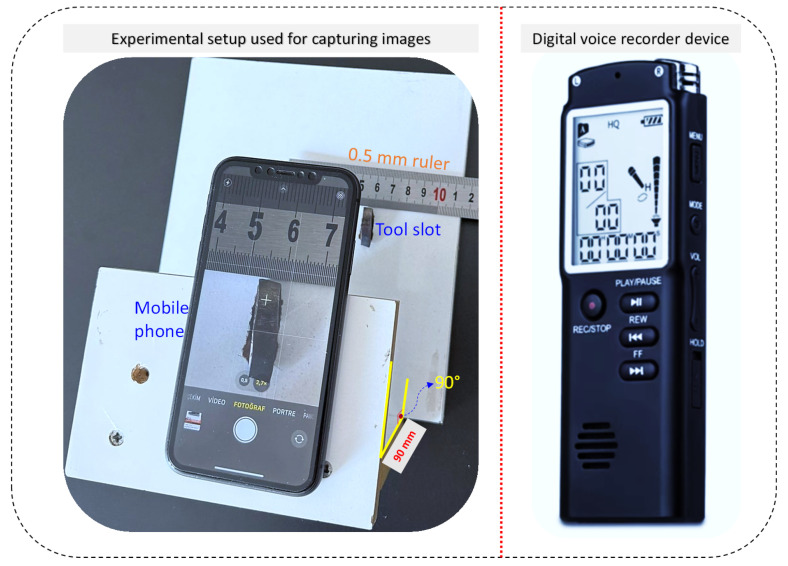
Experimental setup used for capturing images and sound recorder devices.

In this research, a custom imaging setup was developed to ensure consistent geometric conditions during the acquisition of tool-wear photographs for the AISI 316L turning experiments, as shown in Figure 3. An iPhone 15 smartphone (48-MP main camera, f/1.6, sensor-shift OIS) was mounted on a rigid fixture that maintained a fixed 90 mm camera–tool distance and prevented variations in viewing angle. The cutting insert was placed in a machined alignment slot, and a 0.5 mm precision steel ruler was positioned beside the tool edge to provide an internal calibration reference. All images were processed in ImageJ, where pixel dimensions were converted into physical measurements using the embedded scale. This smartphone-based configuration offered a practical and reliable alternative to laboratory-grade optical systems, providing stable imaging geometry and consistent visual quality across all wear levels.

Positioning the sound recorder too close to the cutting zone increases the risk of chip impact and mechanical damage, whereas placing it too far away may introduce unwanted reflections and diffraction effects. For this reason, the recorder must be placed at an appropriate distance that ensures device safety while maintaining high-fidelity acoustic capture [36]. In this research, acoustic data were collected using a digital voice recorder with a Li-ion rechargeable power system, 16 GB internal memory, and a compact form factor (98 mm × 33 mm × 12 mm). The device features an omnidirectional electret condenser microphone and supports high-quality recording modes, including Linear PCM WAV (1536 kbps) and MP3, with a frequency response of 20 Hz–20 kHz. During the AISI 316L machining experiments, the microphone was positioned approximately 5 cm above the tool holder, behind the cutting zone, and opposite the chip-flow direction, ensuring protection from chip impact while preserving signal integrity. Ambient noise around the lathe was also minimized to improve the quality of the recorded sound signals.

## 3. Methods

**Figure 4:**
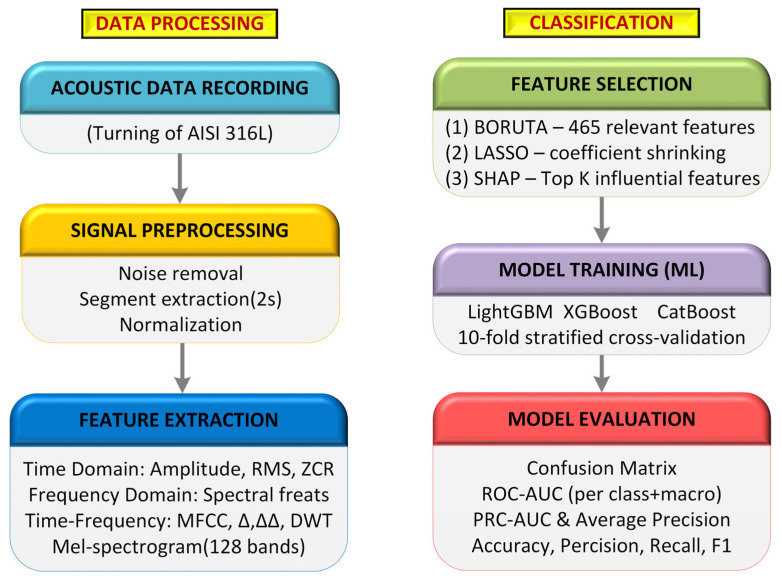
Overall methodological pipeline of the proposed audio-based tool wear classification system.

This section outlines the complete methodological workflow comprising acoustic data acquisition, signal preprocessing, multidomain feature extraction, feature selection, and machine learning–based classification. The pipeline is structured into two primary stages: (1) data processing, which includes acoustic data recording, signal preprocessing, and extraction of time-, frequency-, and time–frequency-domain features; and (2) classification, which involves feature selection using Boruta, LASSO, and SHAP, followed by model training with LightGBM, XGBoost, and CatBoost, and performance evaluation through confusion matrices, ROC–AUC, PRC–AUC, and F1-based metrics. This workflow summarizes the complete procedure applied consistently to AISI 316L datasets. As depicted in Figure 4, the “Overall methodological pipeline of the proposed audio-based tool wear classification system” is provided.

### 3.1. Data Acquisition

**Figure 5:**
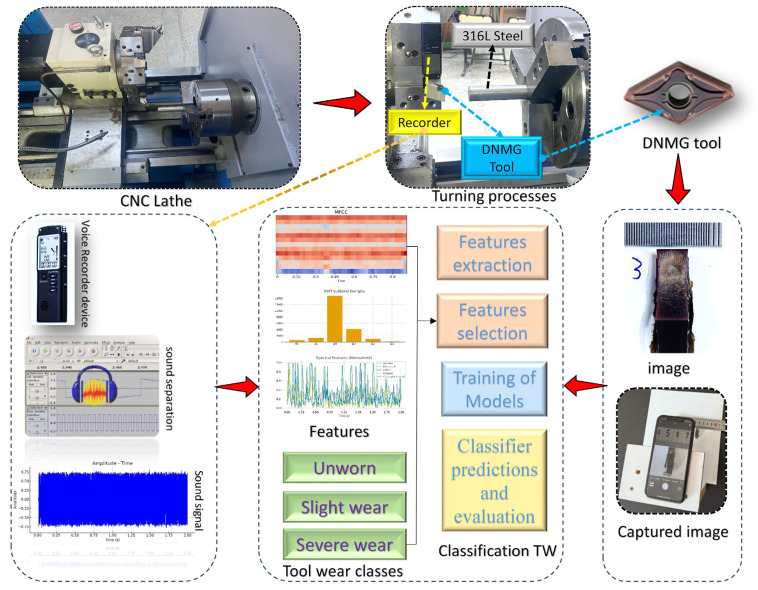
Turning setup, sound acquisition workflow, and preprocessing steps used for TW classification.

As illustrated in Figure 5, tool-wear (TW) evaluation was conducted using acoustic signals recorded during the turning of AISI 316L stainless steel. To ensure a uniform temporal and spectral structure across all recordings, identical cutting parameters were applied in every trial, thereby eliminating machining-condition variability.

Turning operations were performed with a constant feed rate of 0.25 mm/rev, a spindle speed of 1000 rpm, and a depth of cut of 2.0 mm. Each AISI 316L workpiece, prepared as a cylindrical specimen measuring 100 mm in length and 50 mm in diameter, was machined on a CNC lathe using a DNMG-type carbide insert. A two-pass cutting strategy was implemented to generate sufficient wear progression, with each pass contributing incrementally to flank-wear development under identical cutting conditions.

**Figure 6:**
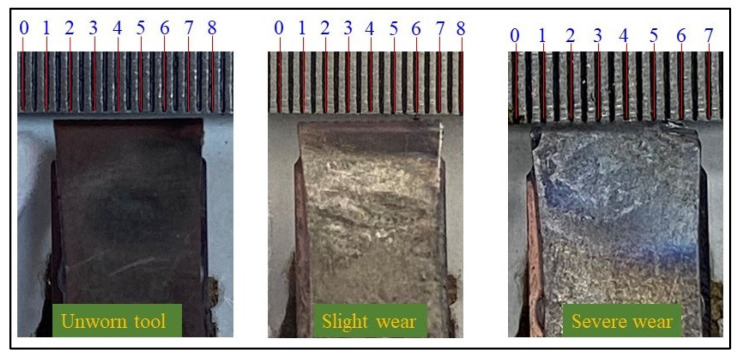
Classification of TW.

Maintaining fixed machining parameters throughout the experimental campaign ensured that variations in the recorded acoustic responses reflected changes in tool wear rather than changes in cutting conditions. After each pass, the tool tip was photographed, and flank wear was measured using image-based evaluation. As shown in Figure 6, the tools were subsequently categorized into three wear states: Unworn, Slight wear, and Severe wear.

Audio segments containing verbal announcements were removed to retain only the cutting sound. The remaining recordings were processed by dividing them into fixed-length 2 s segments, which were used as the fundamental units for model training. Background noise was suppressed through filtering, and each 2 s signal was visualized on a time–amplitude axis before being labeled according to the corresponding tool-wear state. Integrating physical wear measurements with acoustic signal analysis enabled the construction of a reliable dataset for classifying wear conditions in AISI 316L turning, supporting non-contact, rapid, and automated tool-condition monitoring.

### 3.2. Feature Extraction

Acoustic features were extracted directly from each 2 s audio segment using Python 3.9 libraries including Librosa and SciPy. The feature set consisted of time-domain, frequency-domain, and time–frequency-domain descriptors to capture the multi-scale characteristics of tool–workpiece interactions during the turning of AISI 316L.

Time-domain descriptors quantified the amplitude and statistical behavior of the raw signal. These included the root mean square value (RMS), zero-crossing rate (ZCR), mean and maximum absolute amplitude, and the crest factor, defined as the ratio of the peak amplitude to RMS. Higher-order statistics such as skewness and kurtosis were also computed to characterize signal asymmetry and impulsiveness, both of which are sensitive to tool-wear progression [37,38].

Frequency-domain information was extracted from the power spectrum obtained via short-time Fourier transform. The computed descriptors included spectral centroid, spectral bandwidth, and spectral flatness—quantities that describe the distribution and shape of spectral energy. Spectral contrast features, representing the energy difference between spectral peaks and valleys across multiple sub-bands, were also calculated, and summary statistics (mean and standard deviation) were appended for each band [39].

**Figure 7:**
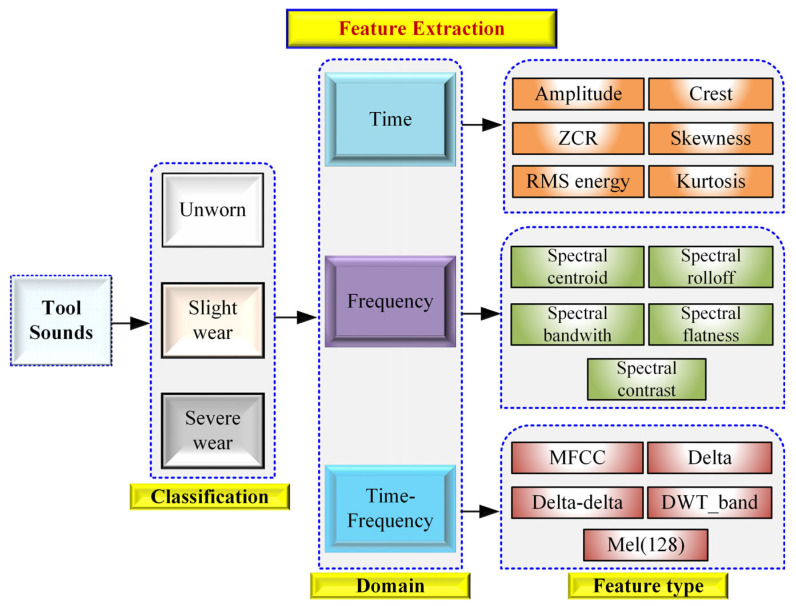
Extraction of statistical features from sounds.

A Mel-spectrogram with 128 Mel bands was generated, and its log-power representation was used to compute 40 Mel-frequency cepstral coefficients (MFCCs). First-order and second-order temporal variations in these coefficients were implicitly incorporated through statistical summaries (mean and standard deviation) across each cepstral dimension. The Mel-spectrogram itself was also summarized using mean and variance descriptors for each of the 128 frequency channels, capturing the global time–frequency energy behavior. Additionally, to characterize transient and non-stationary acoustic patterns associated with chip formation and intermittent friction, discrete wavelet transform (DWT) coefficients were computed using the Daubechies-4 (db4) wavelet. Band-specific wavelet energies were extracted through the dwt_band_features (y) function, providing additional discriminative cues related to high-frequency tool-wear events [40,41]. All features were aggregated into a unified multidomain vector comprising 540 descriptors per segment. Feature names followed the structure implemented in the extraction code (e.g., RMS, ZCR, Crest, Spectral_Centroid, MFCC_1_mean, Mel_5_std, etc.), ensuring consistency between the computational feature pipeline and the machine learning models used in subsequent stages. As shown in Figure 7, the “Extraction of statistical features from sounds” scheme is illustrated.

Table 3 provides a summary of extracted features.

The extracted acoustic features span multiple domains, including time-domain waveforms, Mel-spectrogram representations, MFCCs and their temporal derivatives, discrete wavelet transform (DWT) subband energies, and spectral descriptors, such as centroid, contrast, and flatness. Each representation captures complementary information: temporal structure, spectral envelope variation, transient energy behavior, and frequency-dependent intensity. Together, these multidomain features form a comprehensive basis for discriminating tool-wear states in AISI 316L turning.

Figure 7 depicts the primary multidomain acoustic representations used during feature extraction. The amplitude–time waveform provides information on the temporal structure and intensity fluctuations generated by tool–workpiece interaction. The Mel-spectrogram visualizes the frequency-dependent distribution of acoustic energy, while MFCC representations highlight variations in the spectral envelope associated with different wear states. Additionally, discrete wavelet transform (DWT) subband energies reveal transient phenomena such as impulsive chip–tool contacts and high-frequency bursts. Together, these multidomain representations supply complementary insights into the acoustic behavior of the cutting process and form the basis of the extracted feature set.

**Figure 8:**
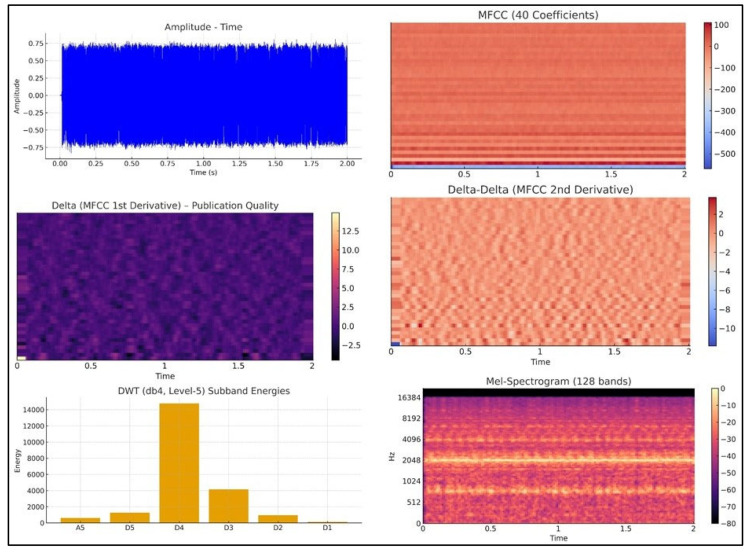
Multidomain acoustic representations (waveform, Mel-spectrogram, MFCCs, and DWT).

Figure 8 presents the statistical and spectral descriptors derived from the recorded audio signals. Time-domain statistics such as RMS, ZCR, amplitude metrics, crest factor, skewness, and kurtosis capture changes in signal magnitude, impulsiveness, and asymmetry that occur as tool wear progresses. Spectral descriptors, including spectral centroid and spectral contrast, characterize shifts in energy distribution and tonal variations in the acoustic response. The temporal evolution of RMS and ZCR also reflects changes in chip formation and cutting stability. These statistical and spectral features provide discriminative information essential for distinguishing different tool-wear states in AISI 316L turning. As shown in Figure 8, “Multidomain acoustic representations (waveform, Mel-spectrogram, MFCCs, DWT)” are displayed. Unlike approaches based solely on Fourier-domain analysis, the present study employs a multidomain feature extraction strategy. Machining sound signals are inherently stochastic and non-stationary, and therefore purely frequency-domain descriptors may not fully capture transient or time-varying wear-related phenomena. For this reason, time-domain statistics, spectral descriptors, MFCC-based cepstral features, Mel-spectrogram representations, and wavelet subband energies were jointly used to provide complementary information about the tool–workpiece interaction.

**Figure 9:**
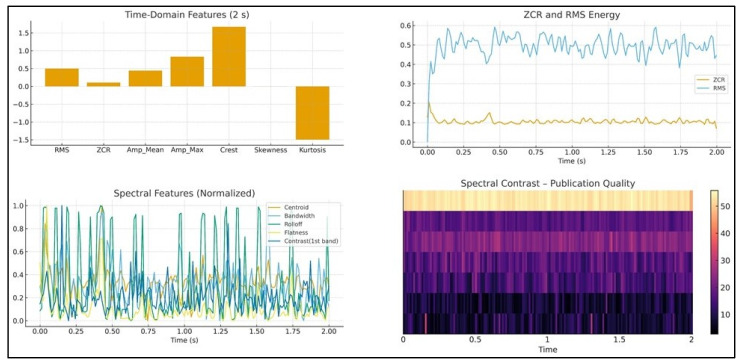
Statistical and spectral audio features (RMS, ZCR, amplitude, crest, and spectral metrics).

Figure 9 depicts the statistical and spectral audio features (RMS, ZCR, amplitude, crest, and spectral metrics).

### 3.3. Feature Selection

**Figure 10:**
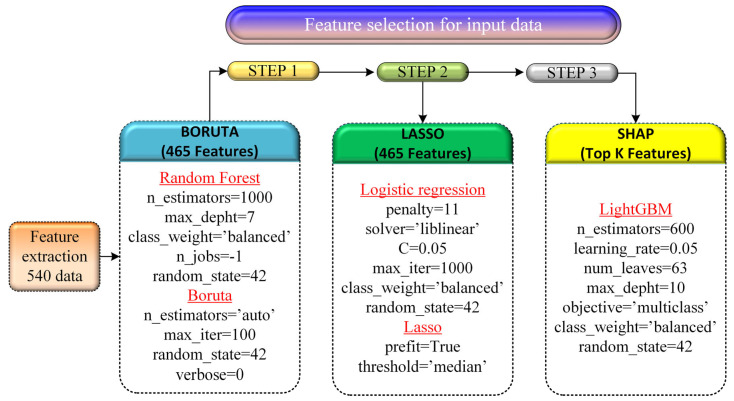
Feature selection.

A three-stage feature selection procedure was implemented to refine the high-dimensional feature set and identify the most discriminative acoustic descriptors for tool-wear classification. In the first stage, the Boruta algorithm was applied using a Random Forest classifier to assess the relevance of each feature. This procedure retained 465 features, indicating their statistical contribution to distinguishing tool-wear states. In the second stage, Least Absolute Shrinkage and Selection Operator (LASSO) regression was employed to perform coefficient shrinkage and evaluate feature sparsity. Since all 465 Boruta-selected features exhibited non-zero or near-threshold coefficients, LASSO did not eliminate additional variables, and the feature count remained at 465. This finding suggests that the Boruta algorithm had already performed an effective relevance filtering of the initial multidomain feature set. In the final stage, model-based interpretability analysis was conducted using SHAP (SHapley Additive exPlanations) values computed from the LightGBM classifier. SHAP identified the top 50 features with the strongest impact on class separation, capturing informative patterns from MFCC statistics, Mel-band structures, wavelet subband energies, and spectral descriptors related to tool–workpiece interaction. This hierarchical selection pipeline ensured a compact yet highly informative feature subset, combining global importance (Boruta), sparsity evaluation (LASSO), and model-specific interpretability (SHAP), thereby enhancing classifier generalization and reducing computational complexity. As depicted in Figure 10, the “Feature selection” scheme is displayed.

### 3.4. Ensemble Learning Classification Models

**Figure 11:**
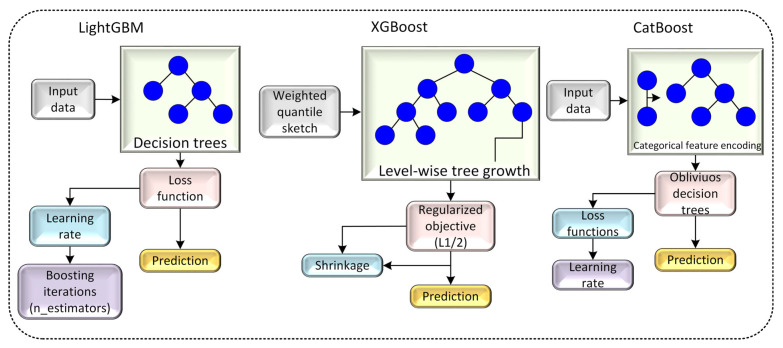
LightGBM, XGBoost and CatBoost classification models.

Three gradient-boosting-based machine learning models—Light Gradient Boosting Machine (LightGBM), Extreme Gradient Boosting (XGBoost), and CatBoost—were employed to classify tool-wear states from the extracted acoustic features. These models were selected due to their strong ability to model nonlinear relationships, handle high-dimensional data, and provide robust performance in signal-based classification tasks. Figure 11 depicts LightGBM, XGBoost and CatBoost classification models.

XGBoost is an advanced GBM algorithm that utilizes first- and second-order gradient statistics and incorporates explicit regularization terms to mitigate overfitting and improve generalization performance. XGBoost uses second-order gradient optimization, regularization terms, and efficient tree-splitting strategies, resulting in improved generalization and reduced overfitting [42]. LightGBM is a faster and more memory-efficient variant of GBM that uses histogram-based DT construction and exploits both first- and second-order derivative information. This strategy provides more accurate and stable optimization while significantly reducing training time and computational cost [43]. LightGBM adopts a histogram-based decision-tree learning approach and leaf-wise growth strategy, enabling fast training and high accuracy when processing the 540-dimensional feature set used in this study. CatBoost utilizes ordered boosting and efficient handling of numerical and categorical variables while minimizing target leakage, making it well-suited for datasets with complex statistical distributions such as machining acoustics [44]. All three models were trained using stratified 10-fold cross-validation to ensure balanced representation of wear states in each fold and reliable estimation of predictive performance. Hyperparameters for each classifier were tuned empirically through systematic parameter exploration and informed by prior studies on acoustic-based tool-condition monitoring. The performance of each model was reported independently without any ensemble or voting mechanism. As depicted in Table 4, the “Models hyperparameters” are given.

### 3.5. K-Fold CV and Classification Metrics

K-fold cross-validation (CV) partitions the dataset into K equally sized folds. In each iteration, one fold is used for testing while the remaining K–1 folds serve as the training set. This process is repeated K times so that every fold is used once as the test set, and the final performance is obtained by averaging the results. This approach provides a robust estimate of model generalization, particularly in studies with limited data [45]. In this work, a 10-fold stratified CV was used to ensure that the class proportions remained consistent across all folds. The dataset was randomly shuffled and divided into ten folds with preserved class distributions. In each iteration, one fold was reserved for testing and the remaining nine folds for training. Averaging the performance across all folds provided a stable and unbiased estimate of classification accuracy while effectively reducing overfitting. Although commonly used values for K include 5 and 10, K = 10 was selected due to its balance between bias and variance for moderate-sized datasets. Model performance for multi-class tool-wear classification was evaluated using accuracy, precision, recall, and F1-score, defined as (Equations (1)–(4)).

(1) $$Accuracy=\frac{TP+TN}{TP+TN+FP+FN}$$

(2) $$Precision=\frac{TP}{TP+FP}$$

(3) $$Recall=\frac{TP}{TP+FN}$$

(4) F
1=2Precision×RecallPrecision+Recall

Here, TP denotes true positives, TN true negatives, FP false positives, and FN false negatives. These metrics jointly quantify classification reliability by measuring correctness (accuracy), positive prediction quality (precision), sensitivity to actual positives (recall), and the harmonic balance between precision and recall (F1-score).

## 4. Results

Before presenting the classification performance, an in-depth analysis of feature relevance was conducted to identify the acoustic descriptors that contributed most to tool-wear discrimination. Feature-importance rankings from LightGBM, XGBoost, and CatBoost, together with SHAP interpretability analysis, were examined to determine which multidomain attributes—such as MFCC statistics, spectral contrast, Mel-band energies, and wavelet components—played dominant roles in predicting the wear state of the AISI 316L cutting tool. These analyses provide insight into the underlying acoustic mechanisms associated with wear progression and establish a transparent basis for interpreting model decisions in subsequent performance evaluations.

### Important Feature and SHAP

**Figure 12:**
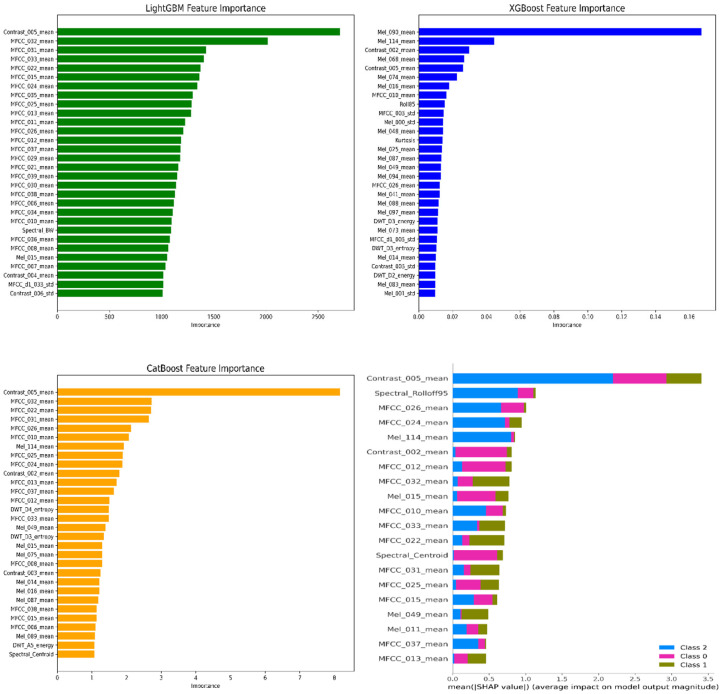
Model-based and model-agnostic feature importance results (LightGBM, XGBoost, CatBoost, and SHAP).

LightGBM identified spectral contrast (Contrast_005_mean) as the most influential feature, followed by several MFCC mean coefficients. The dominance of MFCC- and contrast-based descriptors indicates that variations in the spectral envelope and harmonic structure are highly sensitive to changes in tool–workpiece interaction under different wear states. XGBoost assigned the highest importance to specific Mel-band energies (e.g., Mel_030_mean and Mel_114_mean), suggesting that frequency-localized energy patterns play a critical role in distinguishing wear severity. Contrast and MFCC features also appeared among the top predictors, reflecting the model’s emphasis on both broad spectral shape and finer cepstral variations. CatBoost likewise highlighted spectral contrast and MFCC features as primary contributors, with Contrast_005_mean emerging as the strongest predictor. The consistency of contrast- and MFCC-based descriptors across all three models supports the conclusion that spectral texture and cepstral dynamics carry essential information regarding acoustic signatures of tool wear. SHAP analysis confirmed the dominance of spectral contrast, MFCC means, and Mel-band energies, revealing how each feature influences the predicted class probabilities for Unworn, Slight wear, and Severe wear states. The distribution of SHAP impacts across classes demonstrates that the same features contribute differently depending on wear severity, highlighting their discriminative power in modeling subtle acoustic transitions. As displayed in Figure 12, “Model-based and model-agnostic feature importance results (LightGBM, XGBoost, CatBoost, SHAP)” are presented.

**Figure 13:**
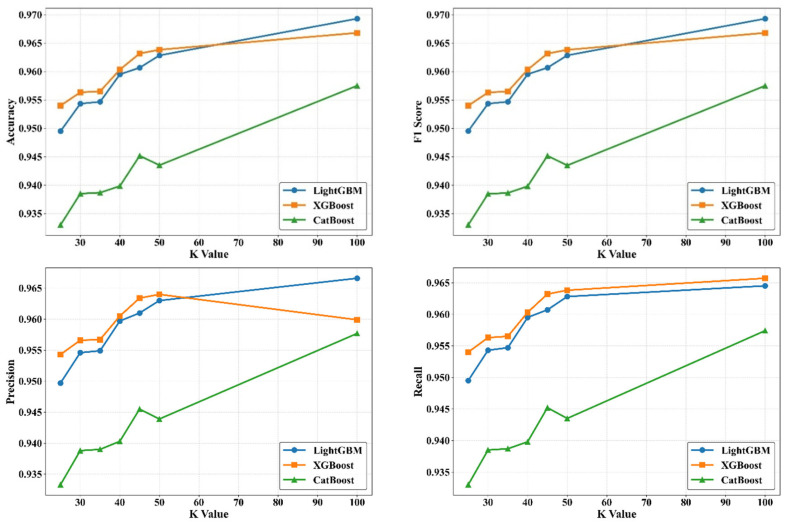
Comparative performance metrics of the classification models.

To evaluate the effect of feature reduction on model performance, the classifiers were retrained using the Top-K features ranked by SHAP importance. K values of 25, 30, 35, 40, 45, 50, and 100 were tested to observe how incremental feature inclusion influences the predictive accuracy of LightGBM, XGBoost, and CatBoost. This analysis provides insight into the optimal dimensionality required for stable tool-wear classification and highlights the sensitivity of each model to feature subset size. In addition, as displayed in Figure 13, “Comparative Performance Metrics of the Classification Models” are provided.

As K increased, both LightGBM and XGBoost exhibited consistent improvements in accuracy, stabilizing near 0.965–0.970 for K ≥ 45. CatBoost showed a similar upward trend but with lower overall accuracy, indicating greater sensitivity to reduced feature dimensionality. These results suggest that approximately 40–50 SHAP-selected features are sufficient for optimal performance in 316L tool-wear classification. The F1-score followed a trend similar to accuracy, with LightGBM and XGBoost achieving their highest values when K ≥ 45. This demonstrates that increasing the number of informative SHAP-ranked features enhances class balance and improves harmonic consistency between precision and recall. CatBoost again showed a gradual rise but maintained lower F1-scores across all K values. Precision increased steadily for LightGBM across all K values, while XGBoost reached its peak around K = 45 before slightly stabilizing. CatBoost showed moderate gains but remained below the other two models, suggesting that its prediction confidence benefits less from incremental feature addition. These observations highlight the strong relationship between spectral-based SHAP features and the discriminative learning capacity of LightGBM and XGBoost. Recall improved as K increased, particularly for LightGBM and XGBoost, which reached values above 0.965 for K ≥ 45. CatBoost demonstrated a slower increase, reflecting its reliance on a larger feature subset to correctly identify minority wear classes. Overall, the results indicate that SHAP-ranking effectively preserves the most informative acoustic descriptors, enabling robust recall performance even with reduced dimensionality. Across all metrics, LightGBM and XGBoost demonstrated superior stability and scalability with respect to the Top-K feature subsets, whereas CatBoost showed increased sensitivity to smaller feature sets. These findings confirm that SHAP-based feature ranking successfully retains the most discriminative acoustic characteristics for accurate and efficient tool-wear classification. Class-wise performance metrics were computed using stratified 10-fold cross-validation to assess the consistency and robustness of each model in distinguishing the three tool-wear states of AISI 316L stainless steel. Table 5 reports the mean and standard deviation of accuracy, precision, recall, and F1-score for CatBoost, LightGBM, and XGBoost across all folds, providing a reliable estimate of their generalization performance under repeated subsampling. Table 5 presents the average class-wise classification performance obtained from 10-fold stratified cross-validation. LightGBM and XGBoost achieved the highest scores across most metrics, particularly for the Unworn and Severe wear classes, while CatBoost showed slightly lower performance and higher variability, especially for the Slight wear class.

**Figure 14:**
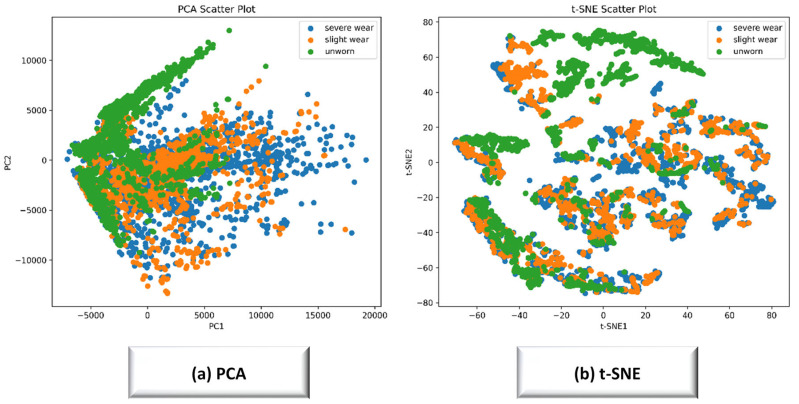
PCA and t-SNE visualization for 316L steel.

The Unworn class yielded the highest accuracy and F1-scores for all models, indicating that early wear conditions produce clearly distinguishable acoustic signatures. LightGBM and XGBoost exhibited strong and stable performance across all wear classes, with mean accuracies exceeding 0.95 for both Severe and Slight wear. CatBoost performed competitively but showed higher fold-to-fold variability, reflected in the larger standard deviations, particularly in the Slight wear class. This class consistently demonstrated lower precision and recall across all models, confirming its transitional acoustic characteristics and inherent difficulty in classification. This behavior is physically consistent with the transitional nature of the Slight wear state, where acoustic characteristics partially overlap with both Unworn and Worn conditions. Overall, the results show that gradient-boosting models—especially LightGBM and XGBoost—provide reliable and high-performing solutions for acoustic-based tool-wear identification in AISI 316L turning. All values were obtained exclusively from 10-fold stratified cross-validation, ensuring statistically robust model comparison. As depicted in Figure 14, “PCA and t-SNE visualization for 316L steel” is provided.

**Figure 15:**
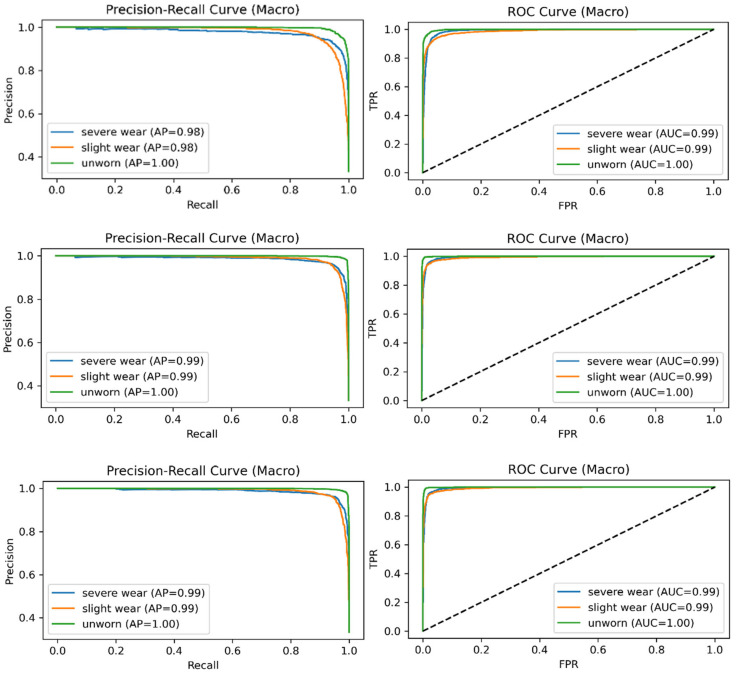
PRC and ROC AUC for 316L steel.

The PCA scatter plot illustrates the distribution of the extracted acoustic features in a reduced two-dimensional space. While the Unworn class forms a partially distinguishable cluster, the Slight wear and Severe wear classes exhibit considerable overlap, reflecting the progressive and continuous nature of acoustic signal changes during tool degradation. The strong mixing between the two Worn classes indicates that their spectral and cepstral characteristics share similar structures in the linear feature space, highlighting the need for more sophisticated nonlinear classifiers to accurately separate these states. The t-SNE visualization reveals clearer separation patterns compared to PCA, demonstrating that nonlinear dimensionality reduction better captures the intrinsic structure of the multidomain acoustic features. Unworn samples form distinct clusters, whereas Slight wear and Severe wear remain partially overlapped but with more visible boundaries than in the PCA plot. This behavior indicates that subtle spectral differences between adjacent wear stages exist but require high-capacity models—such as gradient-boosting classifiers—to be effectively distinguished. Together, the PCA and t-SNE plots confirm that acoustic signatures evolve gradually with tool wear, causing transitional overlap between neighboring wear classes. This supports the class-wise performance behavior observed in the classification results, where Slight wear remains the most challenging class to discriminate. Furthermore, Figure 15 provides separate graphs for “PRC and ROC AUC for 316L steel”.

The precision–recall curves indicate consistently high performance for all models, with AP values ranging from 0.98 to 1.00. The Unworn class exhibits the best separability (AP = 1.00), while Severe and Slight wear also maintain strong precision–recall behavior. The ROC curves further support these findings, showing near-perfect discrimination with AUC values of 0.99 for the Worn classes and 1.00 for the Unworn class. CatBoost provides reliable class separation, although its performance is slightly lower than that of LightGBM and XGBoost. Both LightGBM and XGBoost achieve smooth PRC curves and consistently high AUC values, reflecting robust sensitivity and minimal false-positive rates. Overall, the PRC and ROC results demonstrate that all three boosting models achieve excellent class separability in 316L tool-wear classification, even for the more challenging Slight wear condition.

**Figure 16:**
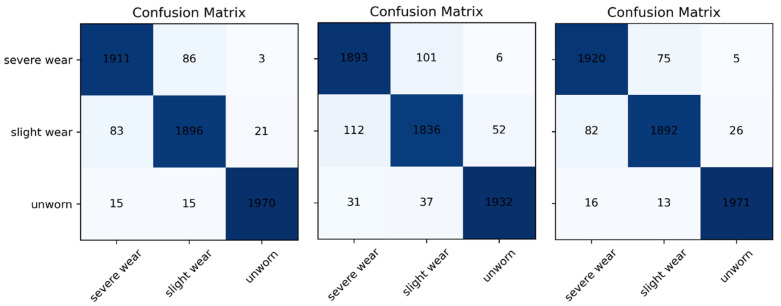
CatBoost, LightGBM, and XGBoost classification results.

All three models demonstrate strong class-discrimination capability, with the highest accuracy consistently observed for the Unworn class. LightGBM and XGBoost yield the best overall separation, showing low misclassification rates across all classes. CatBoost performs well but exhibits slightly higher confusion between Slight wear and Severe wear. Across models, the Slight wear class remains the most challenging to classify, as expected for a transitional wear state whose acoustic characteristics overlap with adjacent classes. Severe wear and Unworn samples, by contrast, show clear boundaries and minimal cross-class confusion, confirming that their acoustic signatures are more distinct. Overall, the confusion matrices support the conclusion that boosted decision-tree models—particularly LightGBM and XGBoost—provide highly reliable recognition of tool-wear conditions in 316L turning. The confusion matrices show that LightGBM and XGBoost achieve the most accurate and balanced classification across the three wear states, with very low misclassification for Severe and Unworn classes. CatBoost also performs well but exhibits slightly higher confusion in the Slight wear class, which is expected due to its transitional acoustic characteristics. Overall, the results confirm that boosted tree models—particularly LightGBM and XGBoost—provide robust wear-state discrimination, while the Slight wear category remains the most challenging due to its intermediate signal patterns. Across all models, the Unworn class shows the highest prediction accuracy, with fewer than 20 misclassified samples in each confusion matrix. LightGBM and XGBoost correctly classify more than 1900 samples in the Severe wear category, demonstrating strong separability for advanced wear states. In contrast, CatBoost exhibits higher confusion in the Slight wear class (e.g., 112 misclassified samples), confirming that this transitional class remains the most challenging to distinguish due to its overlapping acoustic characteristics. Figure 16 visually presents the “CatBoost, LightGBM and XGBoost Classification Results”.

## 5. Discussion

This study introduces a multidomain acoustic-signal framework for classifying tool wear in the turning of AISI 316L stainless steel and positions its findings within the existing literature. Traditional monitoring approaches often rely on force, vibration, temperature, or acoustic-emission sensors, which require additional instrumentation and may interfere with machining conditions [46]. In contrast, Li et al. [22] highlighted the advantages of audible-sound sensors due to their low cost, non-contact nature, and straightforward integration. The high accuracy achieved in this study using only a microphone aligns well with this trend and confirms the viability of sound-based TCM. It should be noted that acoustic-emission sensors typically operate at frequencies beyond the audible range and require specialized mounting and signal-conditioning systems. In contrast, the present study intentionally employed an audible-range microphone to develop a low-cost, non-contact, and easily deployable monitoring solution. The objective was to evaluate whether reliable wear classification could be achieved using only audible sound signals, which offer practical advantages in terms of cost, installation simplicity, and compatibility with existing CNC environments. Previous work on AISI 316L machining has mainly examined how cutting parameters affect machinability, surface integrity, and wear [5,10]. However, the acoustic signatures associated with wear have been less explored. The combination of MFCCs, Mel-band descriptors, spectral features, and wavelet energies used here successfully captured subtle wear-related patterns, consistent with the mechanisms described by Diniz et al. [7]. Compared with noise-reduction and transfer-learning approaches proposed by Zafar et al. [25] and Gao et al. [13], the SHAP-based feature selection applied in this study provided a simpler yet highly effective way to isolate informative descriptors while improving interpretability. The classification accuracy obtained—exceeding 96% with LightGBM and XGBoost—compares favorably with results reported in earlier sound-based and multisensor frameworks. Schueller & Saldaña [14] achieved 92.4% accuracy using combined sound, power, and load signals, while Bhandari [26] reported around 90% using transformer-based acoustic analysis. Similarly, Maia et al. [24] successfully identified wear mechanisms using AE and STFT, but did not match the performance observed here. The transitional Slight wear class remained the most challenging, consistent with the findings of Li et al. [22] and Bhandari [26], who noted strong acoustic overlap among intermediate wear states. Beyond these comparisons, this investigation contributes a compact and efficient framework that integrates multidomain acoustic features with SHAP-enhanced feature selection, enabling both strong predictive performance and clear interpretability. The novel combination of feature groups and explainable boosting models demonstrates that a single audible-sound sensor can rival or surpass several multisensor approaches reported in the literature. These strengths highlight the industrial relevance of the method: the system is low-cost, easy to integrate into CNC environments, non-intrusive, and suitable for real-time monitoring. Its high accuracy suggests strong potential for early-warning functions in production settings, supporting predictive maintenance and smart manufacturing initiatives. Although the proposed framework demonstrated high classification accuracy under controlled conditions, several limitations should be acknowledged. The experiments were conducted using fixed cutting parameters, a single tool–workpiece configuration, and a single audible-range microphone in a laboratory environment to isolate acoustic variations caused by tool-wear progression. While this approach ensured a clear relationship between wear states and acoustic features, it limits direct generalizability to other machining scenarios. Future studies will therefore investigate variable cutting parameters, different tool geometries and coatings, and real industrial environments. In addition, multisensor fusion, deep learning-based feature extraction, time-series wear modeling, and real-time monitoring strategies will be explored to further improve robustness and industrial applicability.

## 6. Conclusions

This study demonstrates that tool wear occurring during the turning of AISI 316L stainless steel can be reliably classified using acoustic signals. The extraction of 540 multidomain features from the time, frequency, and time–frequency domains, combined with a Boruta–LASSO–SHAP feature selection strategy, significantly enhanced the discriminative capability of the classifiers. Among the evaluated models, LightGBM and XGBoost achieved the highest performance, with mean accuracies exceeding 96% and PRC/ROC-AUC values ranging between 0.98 and 1.00. While the Unworn and Severe wear states were distinguished with high stability, the transitional Slight wear class remained more challenging to classify. These findings confirm that gradient-boosting algorithms supported by SHAP-based feature selection constitute a powerful and low-cost combination for sound-based tool-condition monitoring. The research has several limitations, including data collection under fixed cutting conditions, the use of a single tool and workpiece material configuration, and reliance on a single audible-range acoustic sensor. Future research should address these limitations by incorporating variable machining parameters, different tool geometries, multisensor fusion, and deep-learning-based models to enhance industrial applicability and generalizability. Subsequent studies will focus on evaluating model robustness across broader experimental scenarios involving diverse cutting conditions, tool types, and workpiece materials. The integration of multisensor data, advanced deep learning architectures (1D/2D CNN and TCN), and online learning strategies is expected to further strengthen real-time monitoring capabilities. Additionally, the development of noise-robust preprocessing techniques and the direct testing of the proposed framework in real manufacturing environments will be essential steps toward establishing a scalable and industry-ready tool wear monitoring system.

Future research will also focus on time-series wear prediction, real-time monitoring strategies, and the evaluation of computational efficiency and model interpretability. Investigating different tool-coating materials and advanced imaging techniques will further enhance the robustness and industrial applicability of the proposed system.

## Figures and Tables

**Table 1:** Chemical Composition of AISI 316L steel.

Material	Composition Elements: wt.%										
	C	Si	Mn	Cr	Ni	Mo	P	S	Cu	N	Fe
**AISI 316L**	0.011	0.75	0.14	17.1	12.9	2.3	0.007	0.006	0.12	0.067	66.599

**Table 2:** Geometrical parameters of the DNMG150608-MA insert.

Parameter	Value
Name of grade	Carbide
Insert shape code (SC)	Rhombic, 55° included angle
Grade	MC6035
ANSI	DNMG442MA
Insert hand (IH)	Neutral N
Tolerance class insert (TC)	M
Cutting edge length (L) (mm)	15.5
Fixing hole diameter (D1) (mm)	5.16
Inscribed circle (IC) (mm)	12.7
Insert included angle (EPSR)	55°
Thickness (S) (mm)	6.35
Corner radius (RE) (mm)	0.8
Clearance angle (AN)	0°
Chipbreaker type (CBMD)	MA

**Table 3:** Summary of extracted features.

Feature Group	Number of Features
Time-Domain Features	7
Spectral Features	5
Spectral Contrast	14
MFCC (40 coefficients)	80
MFCC Δ (Delta)	80
MFCC ΔΔ (Delta-Delta)	80
Mel-Spectrogram (128 bands)	256
DWT (Wavelet) Features	18
Total Number of Features	540

**Table 4:** Model hyperparameters.

Models	Hyperparameters
LightGBM	n_estimators = 900 learning_rate = 0.05 num_leaves = 63 max_depth = 10 objective = “multiclass” class_weight = “balanced”
XGBoost	n_estimators = 900 learning_rate = 0.05 max_depth = 8 subsample = 0.9 colsample_bytree = 0.9 objective = “multi:softprob” eval_metric = “mlogloss” tree_method = “hist”
CatBoost	iterations = 900 learning_rate = 0.05 depth = 8 loss_function = “MultiClass” verbose = False

**Table 5:** Performance metrics by class for 316L stainless steel (test size = 0.2 and k-10).

Model	Class	Accuracy		Precision		Recall		F1 Score	
		Mean (μ)	Std (σ)	Mean (μ)	Std (σ)	Mean (μ)	Std (σ)	Mean (μ)	Std (σ)
CatBoost	Severe wear	0.9427	0.0080	0.9285	0.0067	0.9415	0.0041	0.9348	0.0074
	Slight wear			0.9265	0.0070	0.9170	0.0057	0.9200	0.0072
	Unworn			0.9705	0.0026	0.9665	0.0024	0.9700	0.0031
LightGBM	Severe wear	0.9583	0.0054	0.9457	0.0051	0.9479	0.0055	0.9465	0.0059
	Slight wear			0.9423	0.0050	0.9410	0.0032	0.9423	0.0047
	Unworn			0.9817	0.0037	0.9803	0.0062	0.9806	0.0050
XGBoost	Severe wear	0.96	0.0041	0.9455	0.0041	0.9498	0.0054	0.9491	0.0060
	Slight wear			0.9492	0.0061	0.9435	0.0028	0.9482	0.0047
	Unworn			0.9822	0.0028	0.9797	0.0037	0.9828	0.0029

## Data Availability

The original contributions presented in this study are included in the article. Further inquiries can be directed to the corresponding authors.
